# Artificial intelligence directed computational protein design: lessons from COVID-19 for pandemic-ready vaccines and antibody therapeutics

**DOI:** 10.3389/jpps.2026.16146

**Published:** 2026-05-08

**Authors:** Rahul Kaushik, Suyong Re

**Affiliations:** Laboratory of In-Silico Design, Artificial Intelligence Center for Health and Biomedical Research, National Institutes of Biomedical Innovation, Health and Nutrition (NIBN), Osaka, Japan

**Keywords:** AI regulations, artificial intelligence, biologics, COVID-19, pandemic preparedness

## Abstract

Artificial intelligence (AI) directed computational protein design has emerged as a transformative force in modern therapeutic discovery, reshaping how vaccines and antibody-based interventions are conceived, optimized, and deployed against emerging infectious diseases. The COVID-19 pandemic served as an unprecedented real-world stress test for these technologies, highlighting their potential to accelerate antigen design, guide antibody optimization, and anticipate viral evolution in near real time. AI driven approaches contributed to faster characterization of viral variants, supported vaccine and broadly neutralizing antibodies developments. Despite the significant contributions, the pandemic also revealed important limitations that must be addressed before such approaches can be relied upon as cornerstones of global preparedness. Challenges related to data bias, model interpretability, experimental validation bottlenecks, and integration with existing regulatory frameworks became increasingly apparent. In several cases, the gap between computational promise and translational readiness underscored the need for closer coupling between *in silico* design, laboratory experimentation, and clinical evaluation. Moreover, the rapid pace of AI innovation often outstripped established regulatory pathways, raising questions about standardization, validation, and long-term safety. This mini review provides a focused overview of recent advances in AI enabled computational protein design, with an emphasis on applications relevant to pandemic response. Drawing on lessons from COVID-19 case studies, it examines translational and regulatory considerations, highlights unresolved controversies, and identifies critical research gaps. Collectively, these insights outline a path toward transitioning AI designed vaccines and antibody therapeutics from reactive emergency tools into proactive, scalable infrastructures for future pandemic preparedness.

## Introduction

The growing frequency of emerging and re-emerging infectious diseases has revealed fundamental limitations in traditional pharmaceutical development pipelines. Conventional vaccine and antibody discovery processes typically rely on sequential experimental screening, optimization, and validation, often requiring years to progress from concept to clinical deployment [1–5]. Such timelines are poorly suited to rapidly spreading pathogens capable of global dissemination within months, particularly in a highly interconnected global landscape. The COVID-19 pandemic starkly illustrated this mismatch, as unprecedented scientific mobilization was required to counter a rapidly evolving viral threat. At the same time, COVID-19 catalyzed the widespread adoption of artificial intelligence (AI)-directed computational approaches as tools for accelerating therapeutic discovery. Advances in data availability, computing infrastructure, and machine learning algorithms converged to enable rapid analysis of viral genomes, structural modeling of antigens, and prioritization of therapeutic candidates at a scale that was not previously feasible. These developments signaled a shift away from purely empirical discovery toward data-driven and design-oriented paradigms [2, 6–10].

AI-enabled protein design represents a fundamental rethinking of how biologics are conceptualized and developed. By leveraging large-scale biological data and machine learning models capable of learning complex sequence–structure–function relationships, these approaches allow systematic exploration of protein space at a scale unattainable by experimental methods alone. Importantly, AI-driven design does not replace experimental science; rather, it reframes it by prioritizing hypotheses, narrowing candidate space, and enabling rational iteration under severe time constraints [3, 5, 11–13]. During the COVID-19 pandemic, AI-driven pipelines contributed to antibody discovery, antigen stabilization, variant impact assessment, and scaffold design, compressing early discovery timelines and enabling rapid iteration in response to viral evolution. These applications spanned multiple methodological paradigms, including sequence-based learning, structure-aware modeling, and generative optimization [7–9, 14–16]. The diversity of successful approaches highlighted both the flexibility of AI-enabled methods and the importance of aligning computational strategies with specific biological questions [1–3, 13, 17].

However, the pandemic also demonstrated that computational acceleration does not automatically translate into clinical or public health impact. Challenges related to reproducibility, model interpretability, experimental validation, data availability, manufacturing scalability, and regulatory acceptance remain significant barriers. Addressing these challenges requires a balanced and critical assessment of what AI-directed protein design can currently deliver, where it falls short, and how it can be responsibly integrated into sustainable pharmaceutical development pipelines.

**FIGURE 1:**
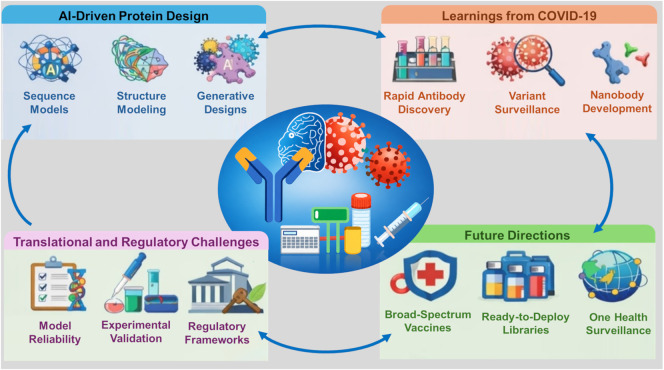
Integrated Framework for AI-Directed Pandemic Preparedness. The schematic provides a multi-quadrant synthesis of the review’s core sections. Upper Left: Technical paradigms in sequence, structure, and generative modeling (referencing Section *AI-directed computational protein design in therapeutics development*; Table 1). Upper Right: Lessons from COVID-19 applications, including rapid antibody discovery and variant surveillance (referencing Section *Methodological paradigms in protein design*). Lower Right: Future trajectories toward broad-spectrum vaccines and “One Health” integration (referencing Section *Translational and regulatory considerations*). Lower Left: Critical translational barriers, including experimental validation and regulatory frameworks (referencing Section *Future directions: Toward pandemic-ready biologics*). The central axis illustrates the convergence of these strategies to accelerate the delivery of next-generation biologics. (Created with BioRender.com).

This mini-review adopts a narrative synthesis approach to evaluate recent advances in AI-directed computational protein design, examining evidence-based lessons from COVID-19 to inform the development of truly pandemic-ready vaccines and antibody therapeutics. As schematically summarized in Figure 1, this framework integrates the methodological, empirical, and regulatory themes explored throughout the following sections to provide a holistic view of the current biodefense landscape.

## Literature selection and methodology

This mini review adopts a narrative synthesis approach to evaluate the integration of artificial intelligence in protein design, focusing on the transition from traditional structural biology to modern generative paradigms. To ensure transparency and reproducibility in our literature selection, we performed systematic searches across PubMed, Google Scholar, and preprint servers (bioRxiv and medRxiv).

### Search strategy and identification

The search strategy utilized targeted strings designed to capture the intersection of machine learning and structural virology, including: AI-directed protein design, machine learning for antibody optimization, generative modeling for SARS-CoV-2 antigens, and *de novo* protein design COVID-19.

### Inclusion and exclusion criteria

Studies were primarily included based on their contribution to the methodological shift toward data-driven paradigms or their direct impact on the accelerated development of vaccines and antibody therapeutics published between 2020 and late 2025. Of the 45 total references cited in this review, 17 are utilized in the Introduction to establish the broader scientific and historical context. The remaining 28 references were specifically selected and analyzed to describe the technical evolution of AI/ML-based strategies in the field of computational protein design. To maintain a focused scope, articles focusing exclusively on clinical outcomes or epidemiological trends without a substantial computational design or structural modeling component were excluded.

### Quality assessment and synthesis

The quality and technical rigor of the selected literature were assessed based on three primary pillars: Peer-review status, clarity of the machine learning architectures employed, and experimental or biophysical validation to ground the reported computational predictions. The insights gathered from this literature selection are synthesized to discuss how these advancements inform the development of truly pandemic-ready biologicals, as schematically summarized in Figure 1.

## AI-directed computational protein design in therapeutics development

Protein-based vaccines and antibody therapeutics rely fundamentally on precise molecular recognition. Traditional experimental approaches, such as phage display, animal immunization, and high-throughput screening are resource-intensive and time-consuming. Computational protein design offers a complementary strategy by enabling rational exploration of vast sequence and structural spaces. Recent advances in artificial intelligence (AI) and computational modeling have transformed both vaccine and antibody therapeutic development, enabling more systematic, scalable, and predictive design workflows [7–9, 11, 18, 19]. Across these applications, sequence-based and structure-informed AI models facilitate the identification, optimization, and functional evaluation of biologics, complementing experimental approaches and reducing reliance on iterative laboratory screening.

### Sequence-based modeling

Sequence-based modeling constitutes the foundation of many AI-driven protein design approaches. By learning statistical patterns from large collections of natural protein sequences, these models capture evolutionary constraints associated with folding, stability, and function. Recent deep learning architectures have enabled sequence models to represent long-range dependencies, contextual relationships, and epistatic interactions that were previously difficult to capture using classical bioinformatics methods [6, 10, 20, 21]. During the COVID-19 pandemic, sequence-based analyses were widely applied to rapidly screen antibody repertoires, identify conserved viral regions, and prioritize targets less susceptible to immune escape [1, 2, 5, 12, 22]. These approaches were particularly valuable in the earliest phases of the outbreak, when structural information was scarce but viral genomic sequences were rapidly accumulating. In this context, sequence-based models enabled rapid hypothesis generation and target prioritization at a time when experimental resources were limited.

Sequence-based approaches have also proven useful for assessing mutational tolerance and identifying regions of functional constraint across viral proteins. Such analyses informed the selection of epitopes likely to remain stable under immune pressure, thereby supporting the design of more durable vaccines and antibody therapeutics [11, 23, 24]. However, these predictions are inherently probabilistic and depend strongly on the diversity and representativeness of training data.

Despite their utility, sequence-only models have inherent limitations. They may fail to fully capture three-dimensional interactions, conformational dynamics, post-translational modifications, and context-dependent effects that strongly influence binding and stability [4, 7, 10, 15, 16, 25]. Consequently, sequence-based predictions are most effective when used as an initial filtering step, guiding subsequent structure-aware analyses rather than serving as standalone decision tools. Recognizing these strengths and limitations is essential for their effective translational application.

### Structure-based modeling

Structure-based modeling leverages three-dimensional information to predict protein folding, binding interactions, and conformational changes that underlie biological function. The availability of high-quality experimental structures, combined with major advances in deep learning–based structure prediction, dramatically expanded the applicability of structure-guided design during the COVID-19 pandemic [4, 7, 11, 19, 21]. Structural models of the SARS-CoV-2 spike protein and its complexes with neutralizing antibodies enabled rational identification of binding hotspots, informed affinity maturation strategies, and supported the design of stabilized antigens [3, 6, 13, 26]. Beyond static representations, structure-based approaches facilitated comparative analysis of antibody binding modes and epitope accessibility, providing mechanistic explanations for differences in neutralization potency. Importantly, structure-based modeling also contributed to understanding why certain antibodies retained activity against emerging variants while others rapidly lost efficacy. By visualizing how specific mutations altered local geometry or electrostatic interactions, these models provided actionable insights for therapeutic redesign and cocktail formulation [18, 22].

Nevertheless, significant uncertainties persist, particularly flexible regions, transient conformations, glycosylated surfaces, and multimeric assemblies. These limitations underscore the need for cautious interpretation of structural predictions and for integrating multiple modeling approaches with experimental validation. In translational settings, structure-based predictions must therefore be viewed as probabilistic guides rather than definitive answers.

### Generative and optimization approaches

Beyond screening existing biological sequences, generative AI models enable *de novo* protein design by learning to sample novel structures and sequences optimized for predefined objectives. These approaches allow for the simultaneous consideration of multi-objective properties, including binding affinity, stability, solubility, and manufacturability [10, 16, 25, 27]. During the COVID-19 response, generative methods were applied to design mini-proteins and optimized antibody scaffolds, demonstrating their potential to expand the functional space of therapeutics beyond evolutionary precedents [13, 15, 26].

#### Concepts and technical architectures

Modern generative efforts have transitioned from purely statistical sequence modeling to structure-first paradigms driven by Denoising Diffusion Probabilistic Models (DDPMs) and Flow-Matching architectures. These models treat protein design as the reversal of a physical corruption process; a model is trained to denoise a random cloud of points into a biologically plausible protein fold. Unlike older autoregressive models that treat proteins as 1D token streams, diffusion-based frameworks such as RFdiffusion, explicitly construct 3D coordinates (backbone Cα atoms and residue frames) throughout the generative sampling process [13, 28]. This allows the AI to navigate the complex equivariant space of protein geometry, ensuring that global symmetry and fold-topology are maintained with atomic-level intent.

#### Evaluation and designability

A critical component of this generative process is the *in silico* validation of hallucinated structures before they reach the wet lab. To ensure biological plausibility, generated backbones are subjected to a Self-Consistency (scRMSD) evaluation. This involves using an independent, high-fidelity structure predictor (e.g., AlphaFold2 or ESMFold) to forward-fold the AI-designed sequence. A design is considered feasible only if the predicted structure matches the original generative 3D backbone with high precision (scRMSD <2 Å).

This iterative workflow, combining generative sampling with structural consistency oracles and biophysical filtering, reinforces the importance of hybrid computational-experimental pipelines in achieving the rapid iteration required for pandemic preparedness [15, 28].

### Immune escape and variant prediction

A defining feature of the COVID-19 pandemic was the rapid emergence of viral variants with altered transmissibility and immune evasion properties. AI-driven models integrating sequence evolution, structural constraints, and mutational scanning data were widely used to assess how specific substitutions might impact antibody binding and vaccine efficacy. These approaches supported real-time evaluation of emerging variants and informed therapeutic updates [1, 2, 5, 26, 29]. Immune escape prediction represents one of the most challenging and consequential applications of AI-directed protein design. While short-term mutational effects can often be captured with reasonable accuracy, forecasting longer-term evolutionary trajectories under immune pressure is considerably more complex. Viral evolution is shaped by trade-offs between transmissibility, immune evasion, replication efficiency, and fitness costs, factors that are difficult to model comprehensively [2, 6, 16, 25, 26].

The COVID-19 experience highlighted the need for cautious interpretation of immune escape predictions and for continuous model updating as new data become available. Rather than serving as definitive forecasts, these models are best viewed as tools for scenario exploration and risk prioritization, informing preparedness strategies rather than dictating fixed design decisions.

### Translational impact

Experience from COVID-19 highlighted that computational approaches have moved beyond supportive roles to become essential tools in pharmaceutical development. While experimental validation remains indispensable, AI-enabled modeling provides a structured framework for narrowing candidate spaces, integrating diverse datasets, and accelerating the discovery and optimization of both vaccines and antibody therapeutics under conditions requiring speed, adaptability, and scalability [2, 3, 11, 12, 26]. Thus, the success of AI driven computational approaches depends heavily on experimental validation, manufacturability considerations, and regulatory pathways, highlighting the importance of integration across the drug development pipeline.

While these applications illustrate the functional scope of AI-directed computational protein design, their performance is ultimately determined by the underlying machine learning paradigms used to represent, learn, and optimize protein sequence–structure–function relationships. Understanding these methodological foundations is essential for interpreting both the strengths and limitations of AI-enabled design pipelines.

## Methodological paradigms in protein design

Methodologically, recent advances in AI-driven protein design are supported by three complementary paradigms: sequence-based protein language models, structure-informed machine learning, and multimodal generative frameworks. These paradigms, supported by the representative computational tools summarized in Table 1, have transitioned the field from descriptive modeling to predictive and generative engineering.

**TABLE 1:** Representative AI/ML-based computational tools available for protein structure prediction, antibody engineering, vaccine antigen design, and immune escape analysis. The key features for each tool include its application, datasets used in training, AI/ML architecture, and over contribution to the field in a sequential manner.

A. Protein structural characterization	
Tool: *AlphaFold3* [7] Access: https://alphafoldserver.com Predicts biomolecular complex structures Protein sequence and structures data Diffusion-based deep learning Structure-guided therapeutic design	Tool: *RoseTTAFold* [8] Access: https://robetta.bakerlab.org Predicts protein structures and complexes Sequences, MSAs, and structural data Three-track neural network Reliable and interpretable structure prediction
Tool: *ColabFold* [14] Access: https://github.com/sokrypton/ColabFold Accelerates protein structure prediction Protein sequences with fast MSA generation AlphaFold2-based deep neural architecture Democratized large-scale structure prediction	Tool: OpenFold [9] Access: https://github.com/aqlaboratory/openfold Reimplements AlphaFold2 for open research Public protein sequence and structure datasets Transformer-based attention architecture Enabled transparency and benchmarking
B. Protein-ligand and binding prediction	
Tool: *DiffDock* [30] Access: https://github.com/gcorso/DiffDock Predicts protein–ligand binding poses Protein–ligand complex datasets Geometric deep learning diffusion models Advanced structure-based drug discovery	Tool: *GNINA* [31] Access: https://github.com/gnina/gnina Scores protein–ligand docking poses Labeled docking and binding affinity data Convolutional neural networks Improved docking-based virtual screening
C. Generative protein design	
Tool: *ProteinMPNN* [15] Access: https://github.com/dauparas/ProteinMPNN Designs sequences on protein backbones Experimentally determined protein structures Graph neural network architecture Robust *de novo* protein sequence design	Tool: *RFDiffusion* [28] https://github.com/RosettaCommons/RFdiffusion Generates protein backbones and binders Protein structural coordinates Denoising diffusion probabilistic models Introduced diffusion-based protein design
Tool: *ProGen2* [25] Access: https://github.com/salesforce/progen Generates functional protein sequences Large-scale protein sequence databases Autoregressive transformer language model Scalable generative protein modeling	Tool: *Chroma* [13] Access: https://github.com/generatebio/chroma Designs functional proteins Protein structures and sequences Diffusion-based generative model Expanded controllable protein design
D. Protein language models (PLMs)	
Tool: *ESM-2* [27] Access: https://github.com/facebookresearch/esm Protein representations and mutation effects Hundreds of millions of protein sequences Transformer-based protein language model Structure, function, and evolution modeling	Tool: *ProtT5* [16] Access: https://huggingface.co/Rostlab Generates embeddings for protein annotation Large unlabeled protein sequence datasets Encoder–decoder transformer architecture Enabled transfer learning in protein biology
E. Variant effect and evolutionary modeling	
Tool: *EVE/EVEscape* [26] Access: https://github.com/OATML/EVE Predicts variant fitness and immune escape Evolutionary sequence variation (MSAs) Bayesian variational autoencoder Linked sequence, disease and immune escape	Tool: *MAVE-NN* [29] Access: https://github.com/fhalab/MLDE Learns genotype–phenotype relationships Deep mutational scanning data Supervised neural networks Interpreted experimental variant effects
F. Immunoinformatics and vaccine design	
Tool: *NetMHCpan/NetMHCIIpan* [24] Access: https://services.healthtech.dtu.dk Predicts peptide–MHC class I and II binding Peptide binding and eluted ligand data Neural network ensemble models State of the art for epitope discovery	Tool: *IEDB Analysis Resource* [23] Access: https://tools.iedb.org/main Epitope prediction and analysis tools Curated immune epitope experimental data Multiple ML and statistical models Platform for immunoinformatics
Tool: *DeepAb* [32] Access:https://github.com/RosettaCommons/DeepAb Antibody variable-region structures Antibody sequences and structures Deep learning with attention mechanisms Improved CDR loop modeling	Tool: *IgFold* [11] Access: https://github.com/Graylab/IgFold Antibody structures prediction Curated antibody structural datasets End-to-end deep learning framework Enabled fast therapeutic antibody modeling

### Sequence-based protein language models (PLMs)

Protein language models (PLMs) apply self-supervised learning to large-scale sequence datasets, treating amino acid sequences as contextual token streams to learn latent representations that encode evolutionary constraints and functional signatures without requiring explicit structural input [15, 25]. Tools such as ESM-2 and ProtT5 exemplify this approach, leveraging transformer-based architectures to predict mutation effects and functional annotations across hundreds of millions of sequences [16, 27]. This scalability enables rapid downstream applications, such as the variant fitness and immune escape predictions performed by EVE/EVEscape, making PLMs particularly valuable for early-stage discovery and tracking rapidly evolving pathogens [26].

### Structure-informed and geometric machine learning

Structure-driven approaches explicitly incorporate three-dimensional information, encoding spatial relationships such as backbone geometry and intermolecular interfaces using graph neural networks (GNNs) or geometric deep learning [7, 8]. AlphaFold3 and RoseTTAFold have established the benchmark for biomolecular complex prediction, enabling structure-guided therapeutic design with high fidelity [7, 8]. These structural insights are extended by specialized docking and scoring tools like DiffDock and GNINA, which utilize diffusion models and convolutional neural networks to predict protein-ligand binding poses [30, 31]. By enforcing physical and spatial constraints, these models achieve high predictive accuracy for tasks dominated by structural determinants, including antibody-antigen interface modeling as seen in platforms like DeepAb and IgFold [11, 32].

### Generative and multimodal integrative frameworks

Building on these predictive foundations, generative AI frameworks now integrate heterogeneous data sources—including sequence, structure, and experimental mutational data—within unified architectures. Denoising diffusion probabilistic models, such as RFdiffusion and Chroma, allow for the *de novo* generation of functional protein backbones and binders tailored to specific viral epitopes [13, 28]. These are often coupled with sequence-design tools like ProteinMPNN or autoregressive models like ProGen2 to ensure fold-stability and biological function [15, 25].

Furthermore, the maturation of immune-informatics resources, such as the IEDB Analysis Resource and NetMHCpan, facilitates the identification of high-affinity epitopes for vaccine antigen design [23, 24]. While these multimodal models improve robustness, experience from the COVID-19 pandemic highlighted the need to validate computational predictions under real-world experimental constraints. Table 1 summarizes the key features, architecture, and contributions of these representative tools. It should be noted that these listings are not intended as a definitive catalogue, as numerous additional methods and resources exist beyond those included to address the complexities of protein engineering.

## Lessons from COVID-19: case studies and applications

The COVID-19 pandemic provided an unprecedented stress test for AI-directed protein design, moving computational workflows from theoretical frameworks to experimental and clinical reality. The following subsections highlight key evidence-based studies that demonstrate the efficacy of these approaches.

### De novo design of hyper-stable miniprotein binders

A pivotal shift in the pandemic response was the move from stabilizing viral proteins to the *de novo* design of synthetic binders. Researchers utilized Rosetta-based and early diffusion models to design small (approx. 55-residue) proteins targeting the SARS-CoV-2 Spike Protein Receptor Binding Domain (RBD) [33]. These designed miniproteins, such as LCB1, demonstrated picomolar neutralization potency, exceeding that of many monoclonal antibodies. Crucially, experimental validation through cryo-EM confirmed that the binders engaged the target exactly as predicted in the computational model [33, 34]. Their superior thermal stability and ability to be produced in *E. coli* provided a scalable alternative to traditional biologics.

### Computational design of self-assembling nanoparticle vaccines

AI played a critical role in the development of multivalent antigens, which provide broader protection than monomeric proteins. The design of the GBP510 (Ruvaxivid) vaccine utilized computational tools to create a self-assembling protein nanoparticle (icosahedral symmetry) decorated with 60 copies of the SARS-CoV-2 RBD [35, 36]. Clinical trial data showed that this AI-scaffolded design elicited significantly higher neutralizing antibody titters compared to the monomeric RBD or mRNA-based benchmarks in certain populations. This provides concrete evidence that computational geometry can directly enhance the human immune response.

### Generative AI for rapid antibody optimization

As variants like Omicron emerged, AI was deployed to future-proof antibody therapeutics by identifying conserved epitopes. Studies employing Deep Mutational Scanning (DMS) data combined with Graph Neural Networks (GNNs) allowed for the rapid optimization of antibodies like Sotrovimab derivatives. By predicting escape trajectories, researchers were able to engineer antibodies that maintained high-affinity binding across multiple variants of concern [37–39]. Experimental assays confirmed that these AI-optimized candidates retained neutralization against the BA.2 and BA.5 subvariants when many traditional antibodies failed [40, 41].

Importantly, these case studies reveal that the success of AI-enabled approaches depends not only on algorithms but also on high-quality data availability and experimental throughput. Regions with limited sequencing or experimental capacity were less able to benefit from these advances, highlighting equity and infrastructure considerations that must be addressed for future pandemic preparedness.

## Translational and regulatory considerations

Despite their promise, AI-enabled protein design approaches face significant challenges in pharmaceutical translation. Regulatory acceptance requires transparent, reproducible, and validated computational models, as well as clear documentation of how predictions are generated and used. During COVID-19, variability in model predictions underscored the need for standardized benchmarking, reporting practices, and version control [3, 12, 42].

Integration with experimental pipelines proved essential for successful translation. Hybrid workflows combining *in silico* screening with targeted wet-lab validation reduced attrition and improved confidence in AI-generated candidates. Such integration also facilitated early assessment of manufacturability, stability, and developability, factors that are critical for clinical success but often overlooked in purely computational studies [1–3, 6, 12, 42].

Current regulatory frameworks were not designed with AI-driven discovery in mind. While the pandemic prompted limited flexibility in review processes, long-term adoption of AI-designed biologics will require clearer guidance on model validation, lifecycle management, update policies, and post-approval monitoring. Developing such frameworks will be essential to balance innovation with patient safety.

## Future directions: toward pandemic-ready biologics

Preparing for future pandemics, including hypothetical Disease X scenarios, requires a shift from reactive therapeutic development to anticipatory design. Pre-emptive development of broad-spectrum vaccines and antibodies targeting conserved viral elements represents a central goal of this paradigm, offering the potential for baseline protection against entire viral families [1, 19, 24, 26, 43]. Curated libraries of ready-to-deploy biologics could further accelerate response timelines by enabling rapid selection and adaptation of pre-validated candidates. Such libraries would need to be continuously updated as new data emerge, integrating insights from viral surveillance, structural biology, and immunology. Integration of One Health data spanning human, animal, and environmental surveillance will be critical for identifying zoonotic risks before spillover occurs [1, 2, 4, 44, 45]. Coupled with global data-sharing platforms that enable real-time model updating, these approaches could support coordinated and proactive responses to emerging threats. Realizing this vision will require sustained investment in data infrastructure, governance frameworks, and international collaboration.

## Strengths, limitations, and challenges

The rapid evolution of AI-directed protein design necessitates a critical appraisal of the current methodological landscape and the scope of this synthesis. The primary strength of this mini review is its focus on the transition from theoretical *in silico* models to experimentally validated evidence-based outcomes, capturing the shift toward generative paradigms through late 2025. However, this study is limited by the inherent black-box nature of many deep learning architectures, which complicates a purely mechanistic interpretation of design successes. Furthermore, the rapid pace of the field means that some proprietary models may lack the open-source data required for a full technical audit.

### High computational costs and resource disparity

A significant challenge in modern protein design is the escalating computational cost associated with training and deploying state-of-the-art models. The high-fidelity diffusion models and large-scale molecular dynamics (MD) simulations require massive GPU clusters, creating a compute divide that favors well-funded institutions. Further, the energy requirements for these iterative design cycles raise concerns regarding the environmental sustainability of always-on pandemic surveillance systems.

### Data privacy and biosecurity

As AI models are increasingly trained on vast repositories of viral sequences and human immune repertoires, data privacy and biosecurity have emerged as paramount concerns. The use of patient-derived monoclonal antibody data requires rigorous anonymization and secure handling to prevent the exposure of sensitive clinical information. Further, there is an ongoing debate regarding the dual-use potential of generative AI, where the same tools used to design vaccines could theoretically be repurposed to enhance viral fitness or escape, necessitating robust international governance and red-teaming protocols.

### Experimental bottleneck

The primary operational challenge remains the disparity between the speed of AI-generated designs and the throughput of wet-lab validation. Even with high-affinity predictions, the design-build-test cycle is often slowed by traditional synthesis and purification timelines, highlighting the need for more integrated, automated bio-foundries to achieve true pandemic readiness.

Addressing these integrated challenges, from high computational overhead to the ethical management of biological data, remains a prerequisite for the maturation of the field. Only by harmonizing rapid algorithmic innovation with sustainable infrastructure and robust biosecurity protocols can AI-directed design transition from an experimental urgency into a dependable pillar of public health. Ultimately, navigating these multifaceted limitations will define the trajectory of the next-generation of pandemic-ready therapeutics.

## Conclusion

AI-directed computational protein design has fundamentally reshaped the landscape of vaccine and antibody development, a shift catalyzed by the urgent demands of the COVID-19 pandemic. This mini review has highlighted how the transition from reactive to proactive design paradigms, moving from the stabilization of viral antigens to the *de novo* generation of hyper-stable binders, has moved computational workflows into the realm of clinical reality. While the potential of these technologies to accelerate discovery and expand therapeutic breadth is well-established, the field remains balanced by critical considerations regarding model interpretability and predictive reliability. The pandemic underscored that the success of AI-driven biologics is inextricably linked to the availability of high-quality experimental data and the presence of standardized validation frameworks.

Ultimately, the maturation of AI-enabled protein design depends on its integration as a supportive component of global pandemic preparedness, complementing established therapeutic development frameworks rather than replacing them. By grounding computational innovation in rigorous experimental validation and transparent regulatory oversight, these technologies offer a robust path toward ensuring that the global scientific community is better equipped for future infectious disease challenges.
